# Microcapillary Reactors via Coaxial Electrospinning: Fabrication of Small Poly(Acrylic Acid) Gel Beads and Thin Threads of Biological Cell Dimensions

**DOI:** 10.3390/gels7020037

**Published:** 2021-03-30

**Authors:** Susan K. Kozawa, Audrey Lord, Jonah J. Scott-McKean, Anne Y. Walker, Alberto C. S. Costa, Gary E. Wnek

**Affiliations:** 1 Department of Macromolecular Science and Engineering, Case Western Reserve University, Cleveland, OH 44106, USA; skk61@case.edu (S.K.K.); ael84@case.edu (A.L.); ayw2@case.edu (A.Y.W.); 2 Departments of Pediatrics and Psychiatry, Case Western Reserve University, Cleveland, OH 44106, USA; jonah.scott-mckean@case.edu (J.J.S.-M.); alberto.costa@case.edu (A.C.S.C.)

**Keywords:** polyelectrolyte gels, electrospinning, polyelectrolyte threads, bio-mimicking

## Abstract

Poly(acrylic acid) (PAA) bulk gels and threads, typically derived via free-radical polymerization, are of interest as anionic polyelectrolyte mimics of cellular cytosol and as models for early protocells. The thread dimensions have been limited by the diameters of readily-available glass or plastic capillaries, and threads with diameters of less than 50 µm have been difficult to achieve. Here, we report a useful approach for achieving crosslinked, partially neutralized PAA, namely poly(acrylate), gel threads with diameters of a few microns when dry. This technique utilizes coaxial electrospinning to effectively produce capillaries (shells) of polystyrene loaded with a gel-forming precursor mixture composed of 3 M acrylic acid, methylene-bisacrylamide, potassium persulfate and 2.2 M NaOH in the core, followed by thermally-induced polymerization and then the removal of the polystyrene shell. Relatively long (up to 5 mm), continuous PAA threads with thicknesses of 5–15 µm are readily obtained, along with a multitude of PAA gel particles, which result from the occasional break-up of the fluid core prior to gel formation during the electrospinning process. The threads and beads are of the sizes of interest to model ancient protocells, certain functional aspects of excitable cells, such as myocytes and neurons, and various membraneless organelles.

## 1. Introduction

Fumio Oosawa noted that “Biological systems such as muscle, membranes, and protoplasm in general may be regarded as organized polyelectrolytes [1].” Indeed, the cellular cytosol is rich in anionic polyelectrolytes, which make up the bulk of the cytoskeletal framework [2]. In particular, poly(acrylate) gels have enjoyed attention as mimics of the cytosol protein milieu. For example, Tasaki [3,4] has proposed the unorthodox hypothesis that the electrophysiological processes known as neuronal excitation and conduction are fundamentally manifestations of abrupt phase transitions of the cytoskeleton in the cortical gel layer of the axon, which could be mimicked using synthetic polyanionic hydrogels. A particular phenomenon of interest is the existence of abrupt dimensional changes associated with ion-exchange processes, specifically between Ca^2+^ and monovalent cations such as Na^+^.

Although modern cells use a variety of voltage and ligand gated channels as well as complex networks of signaling pathways to precisely modulate excitability and signal transduction, it is possible that protocells of primitive organisms might have had been reliant on simpler mechanisms dependent primarily on the basic properties of intracellular polyanionic hydrogels, similar to those seen in poly(acrylate) gels. In this context, poly(acrylate) gels might also serve as models for some of many classes of polyanionic hydrogel cellular membraneless organelles that have become the focus of many recent biophysical studies [5,6,7,8].

In order to gain a deeper understanding of the role that polyelectrolytes play in cellular excitability and signal transduction, there is interest in suitable synthetic mimics of the cytoskeleton with bulk cellular dimensions, i.e., ca. 5–10 µm in diameter, for common spherical cells, being thin (several µm in diameter) and long (several cm or more), similar to neurites. Ideally, such synthetic cytoskeleton mimics should be capable of exhibiting cell-like phenomena, such as volume changes depending on the exterior solute composition, the generation of electrical potentials between the synthetic material and the bathing medium, and motion resulting from electrical or mechanical stimulation [9,10].

The polyacrylate gels typically studied are synthesized by thermal free-radical polymerization using a recipe comprising 3 M acrylic acid, methylene-bisacrylamide (crosslinker), potassium persulfate (initiator) and 2.2 M NaOH within plastic or glass capillaries. The products, termed gel threads, are extruded from the ends of the capillaries upon swelling in water. A limitation of this approach is that the thread width is determined by the internal diameters of readily available capillaries. The thinnest threads reported by Tasaki [3,4] were prepared in capillaries with an internal diameter of 0.05 mm (50 µm). Upon swelling and extrusion, gel threads with widths of about 200–400 µm are obtained, which are well beyond most neurite dimensions and vastly exceed those of cytoskeletal fibers. In contrast, thin (ca. 0.5–5 µm) fibers of poly(acrylic acid) (PAA), followed by neutralization to the poly(acrylate) derivative, are readily fabricated using electrospinning followed by crosslinking and isolation [11]. However, the post-crosslinking of high molecular weight PAA fibers is fundamentally different from the synthesis of the gel threads described above.

We sought a method to replicate the gel thread preparation on the size scale offered by electrospun fibers, and report here a simple and useful approach for generating poly(acrylate) gels threads from pre-gel compositions trapped within cores of hollow polystyrene (PS) fibers using coaxial electrospinning [12,13,14,15]. Thermal polymerization to form the PAA gel is carried out inside the PS fiber shells, followed by the dissolution of the PS. The resulting gel threads, after swelling in water, have diameters of several microns to tens of microns, depending upon the distance between the electrified jet ejection point and the collector. In addition, due to the occasional break-up of pre-gel cores in PS shells during electrospinning, presumably via a Rayleigh instability of the rather fluid core, it is possible to simultaneously isolate gel droplets along with multi-centimeter-long, thin threads. The relative proportions of threads vs. beads are very dependent on the solvent composition. For example, threads are formed in greater abundance with polystyrene cores spun from 1:1 DMF:chloroform, with a preference for large droplets with PS in chloroform. The resulting gel threads have diameters of several microns after swelling in water. In addition, due to the occasional break-up of pre-gel cores in PS shells during electrospinning, it is possible to simultaneously isolate spherical gels of 1–5 µm diameter along with multi-millimeter-long, thin threads. The use of electrospun shells as a confined postspinning reaction space is relatively unexplored, with the most notable previous example being the work of Reddy et al. [14] on the photochemical curing of liquid epoxy prepolymers, wherein the key points of successful coaxial electrospinning with liquid cores were outlined.

In the present study, we used optical microscopy to quantify the contraction and expansion of thin gel threads stimulated with Ca^2+^ and ATP, respectively. In addition, we performed measurements of static electrical potentials to further characterize these gel threads.

## 2. Results and Discussion

### 2.1. Electrospinning Conditions That Favor Long PAA Threads

**Figure 1:**
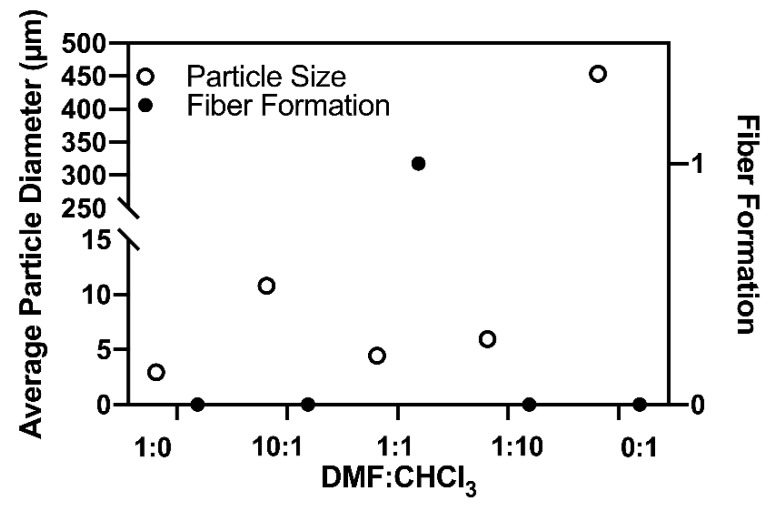
Average particle diameter and fiber formation of the varying DMF:CHCl_3_ spinning solutions.

**Figure 2:**
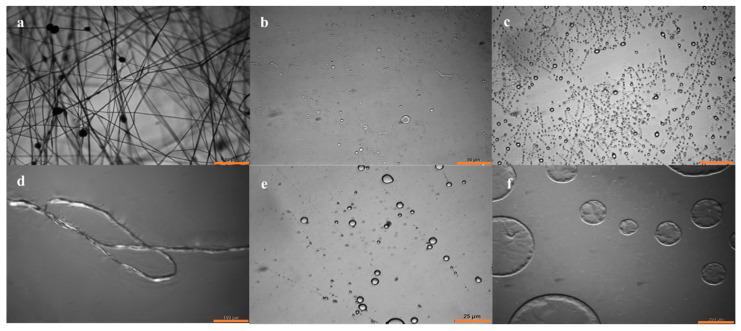
Optical microscopy images of (**a**) PAA spun with PS, scale bar 50 µm; (**b**) PAA particles with PS removed spun with DMF:CHCl_3_ 1:0, scale bar 50 µm; (**c**) PAA particles spun with PS removed with DMF:CHCl_3_ 10:1, scale bar 25 µm; (**d**) PAA thread spun with PS removed with DMF:CHCl_3_ 1:1, scale bar 100 µm; (**e**) PAA particles spun with PS removed with DMF:CHCl_3_ 1:10, scale bar 25 µm; and (**f**) PAA droplets spun with PS removed with DMF:CHCl_3_ 0:1, scale bar 200 µm.

Attention was directed toward conditions that would favor the formation of continuous electrospun coaxial fibers and would moreover minimize pre-gel solution break-up within the PS shells in order to ultimately afford isolatable, crosslinked PAA threads. Therefore, two fluid instabilities needed to be mitigated, namely (1) the break-up of the composite core-shell jet into droplets leading to fiber electrospinning rather than undesired electrospraying, and (2) the break-up of the core pre-gel liquid within an electrospun core-shell fiber. The pre-gel composition was kept constant in all experiments, with the PS solution composition comprising of DMF/chloroform adjusted in various solvent ratios. PS was varied from 10–20% DMF at 10–15 kV in coaxial electrospinning to determine the resulting formation, as seen in Table 1, deeming 10% PS at 12 kV to be the most stable for fiber formation. It was empirically found that a DMF:CHCl_3_ ratio of 1:10 produced the most stable continuous fibers, while the best PAA threads in terms of overall length and frequency of threads vs. droplets within PS cores were derived from PS in 1:1 DMF:CHCl_3_. Thread thicknesses ranged from 2 to 17 µm (average of 5–7 µm), with lengths frequently exceeding 1–2 cm. Gel beads are differentiated between droplets and particles in Table 1, and trends are summarized in Figure 1. For example, droplets lead to large disc-shaped beads largely shaped by gravity, with particles shaped into microspheres by the conventional electrospinning beading process. Droplets (gel beads after crosslinking) were consistently favored when only CHCl_3_ was employed as the solvent for PS. Here, very large droplets formed as the result of unstable jet break-up without the formation of stable threads. We note that the selected sheath polymer-solvent system should be electrospinnable by itself in order to contribute to the core-sheath structure formation [13], and in our hands PS in CHCl_3_ only does not electrospin well. Examples of the range of behavior discussed are shown in Figure 2.

To achieve a stable thread formation via the suppression of the break-up of the composite core-shell jet into electrospray droplets, the amplification of disturbances on the jet should be minimized. Fundamentally, liquid threads are unstable to distortions with a wavelength larger than the circumference of the thread. It is known that the growth rate of capillary deformations depends on the properties of the liquids, mainly the surface tensions (*γ*) and viscosities (*η*). More stable and therefore longer jets are favored when *γ* is reduced and *η* is increased [13]. A quantitative analysis of the conditions that favor threads vs. beads within the fibers is difficult to perform due to the potential interplay of multiple contributing factors, including the conductivities, dielectric constants and viscosities of core and shell liquids, and the interfacial tension between the two. Regarding viscosity differences, a proposal by Tomotika [16], succinctly summarized by Elmendorp [17], posits that the dominant growth rate for sinusoidal disturbances for threads of a viscous material in a matrix of another depends in part on the ratio of thread (η_t_) to matrix (η_m_) viscosities. Specifically, η_t/_η_m_ << 1 favors a thread break-up into droplets. It is known that the viscosity of a 10 wt% solution of PS of M_n_ ca. 120 kD is about 35% lower than a comparable solution in chloroform [18], and perhaps a lower shell viscosity (effectively η_m_) results in a shift toward more stable core threads. The rapid mixing of water and acrylic acid from the core would be expected to further reduce η_m_ due to the collapse of PS coils as the result of a decrease in the solvent quality, thus encouraging the formation of rather long core threads.

Further, it is reasonable to assume that the ionic conductivity of the core containing acrylic acid and NaOH should be considerably greater than that of the PS shell, especially when the solvent composition is rich in chloroform. The case of 1:1 DMF/CHCl_3_, which we find yields the best gel threads, is further complicated by the miscibility of DMF and water, which could cause at least a partial precipitation of PS at the core-shell fluid interface. We suggest that this possibility, coupled with the greater viscosity of the PS-solvent shell along with an anticipated low interfacial tension between the aqueous core and DMF-containing shell, can assist in stabilizing the fluid core against break-up into droplets. These effects would be lessened when only CHCl_3_ is employed as the PS solvent, a situation that favors the formation of large droplets.

As noted earlier, PAA threads made from the DMF:CHCl_3_ 1:1 ratio produced the most consistent fiber formation, ranging in diameter from 2 to 17 µm (average diameter of 5–7 µm). The PS layer is removed thoroughly using a separation funnel. The fibers and particles are shaken vigorously with toluene, and water is added, allowing the PAA to move towards the aqueous layer. As the solution settles, the cleaned PAA moves towards the aqueous layer and is collected. FT-IR spectra (not shown) of crosslinked PAA particles after immersion in acetone for several hours indicate the absence of aromatic group vibrations (ca. 1604 cm^−1^ and others) and the successful removal of PS shells.

### 2.2. Swelling and Deswelling of PAA Threads

**Figure 3:**
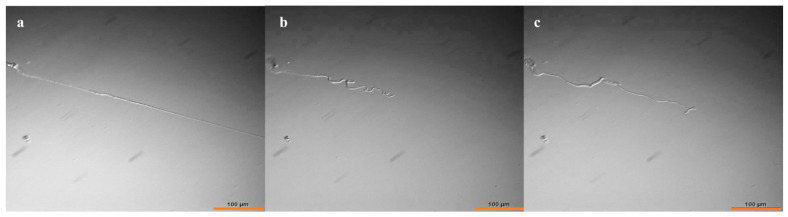
Optical microscopy images of (**a**) PAA(Na^+^) thread expanded in water, (**b**) PAA thread contracted with CaCl_2_ and (**c**) PAA thread re-expanded with ATP. Scale bar 100 µm.

The resulting PAA was swollen and deswollen using varying solutions to demonstrate its anticipated properties as an anionic polyelectrolyte and to loosely mimic the contraction and expansion of muscle. The PAA was equilibrated in each solution prior to imaging, resulting in a swelling/deswelling effect based on ion exchange. Figure 3 shows an optical micrograph of an isolated PAA thread (Na^+^ form) equilibrated in DI water and then treated with 500 mM CaCl_2_. A significant axial contraction (ca. 44%) is observed (Figure 3b), as expected from studies on similar gels of much larger dimensions [3,4,19,20]. The chelation of Ca^2+^ was expected to at least partially restore the original dimensions of the thread. Toward that end, we employed ATP as a Ca^2+^ chelator [21], and indeed a partial reswelling (ca. 75%) of the original thread length was observed (Figure 3c). A full re-extension to the initial length was of course not expected for entropic reasons. However, the fact that two key, biologically relevant species can elicit motion in thin PAA threads is especially interesting for the design of synthetic cytoskeletons for artificial cells. The thin PAA threads are expected to facilitate the kinetics of these ion-exchange processes, and this is a subject of continuing studies that include the influence of the bathing salt solution type and concentration on kinetics. Admittedly, the free radical polymerization of these gels leads to heterogeneities in the gel network, which can lead to asymmetric bending that is characteristic for these gels.

### 2.3. Electrical Potentials of PAA Threads

**Figure 4:**
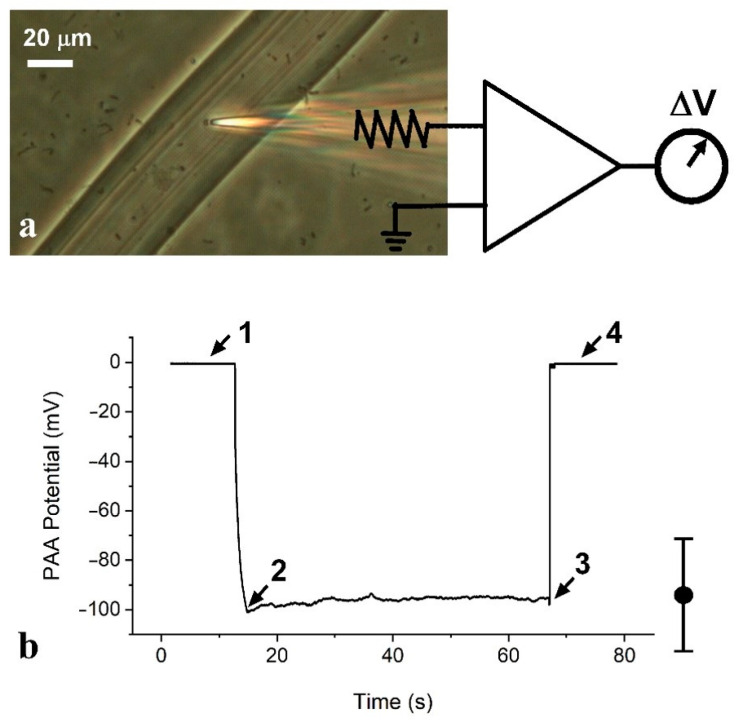
Recording of one measurement from the PAA thread in a 16-mM KCl solution: (**a**) recording electrode tip penetration into a gel thread; (**b**) potential upon impalement of thread (2) followed by withdrawal (3). The mean of the electrical potential difference (ΔV) obtained from 10 measurements was −93.6 mV, with a standard deviation of ±22.8 mV.

It is known that PAA gels exhibit negative electrical potentials (relative to the bulk external solution) with a sign and magnitude that is surprisingly similar to living cells [22], albeit at a low bathing ionic strength in our experiments. To further characterize the properties of our PAA gel threads, electrical potentials were measured with threads after immersion in 16 mM KCl for 24 h using standard electrophysiological methods. The procedure for physically measuring the electrical potential difference (ΔV) between the bulk solution and the interior of the PAA threads involved inserting the recording micropipette into the PAA threads under simultaneous visual guidance through an inverted microscope and electrical potential monitoring with a differential amplifier (Figure 4a). Because the PAA threads adhered to the glass bottom of the recording chamber, they did not need to be mechanically held in place. Voltage recordings started with the micropipette outside the PAA thread, which was used as the zero-millivolt baseline, measured in relation to a ground Ag/AgCl electrode in the bathing solution (Figure 4b; arrow “1”). When the micropipette was lowered into the PAA, we could observe the penetration of the fiber both optically and by the simultaneous negative change in the pipette-tip potential (Figure 4b; arrow “2”). This negative potential difference measurement was robust and quickly stabilized at several tens of negative millivolts in relation to the reference potential in the bathing solution (Figure 4b; arrow “3”). Importantly, an additional penetration of the micropipette beyond the initial level produced no alteration in this measured negative internal potential. When the micropipette was removed from inside the thread, the recorded potential quickly returned to the reference, ground level (Figure 4b; arrow “4”). The optical visualization of the track that was left after the pipette was removed (not shown) provided additional confirmation of the micropipette’s penetration of the PAA thread. This protocol allowed for very stable and reproducible recordings across dozens of PAA threads. Ten measurements from a thread 15 µm in width produced a mean potential difference (ΔV) of −93.6 ± 22.8 mV (arithmetic mean ± standard deviation). 

The measured potentials are likely to be at least in part a Donnan potential, φ_D_, which is related to the ratio of the activities of mobile ions in the bathing solution and inside the gel. It has been noted that a true correlation between calculated and measured Donnan potentials is highly dependent on probe electrode dimensions [23], which we did not explore here, although the potentials measured for polyacrylate gel threads are highly stable and within a range that is consistent with related studies on much larger bulk polyacrylate gels [24].

## 3. Conclusions

The coaxial electrospinning process is advantageous for encapsulating polyacrylate pre-gel solutions, which can subsequently be gelled by thermal initiation within a solvent-extractable shell. It is proposed that these gels may be useful as cytoskeletal mimics for artificial cells or models for certain types of membraneless cytoplasm organelles. Additional potential applications include matrices for the immobilization of enzymes and biological cells.

## 4. Materials and Methods

Polystyrene (PS) (MW 130,000 Sigma Aldrich, St. Louis, MO, USA) formed the coaxial electrospinning outer sheath solution as a 10 wt% polymer solution with a solvent combination of DMF:CHCl_3_ at 1:0, 10:1, 1:1, 1:10 and 0:1 ratios, respectively (Fisher, Pittsburg, PA, USA). The inner core is composed of a poly acrylic acid (PAA) monomer solution; 3 M acrylic acid (AAc), 2.2 M sodium hydroxide (NaOH), 5 mM *N,N* methylene-bisacrylamide (“bis”) and 3 mM potassium persulfate (KPS) (Millipore Sigma, Munich, Germany) were added to distilled deionized water, a formulation adapted from Tasaki [3,4]. 

**Figure 5:**
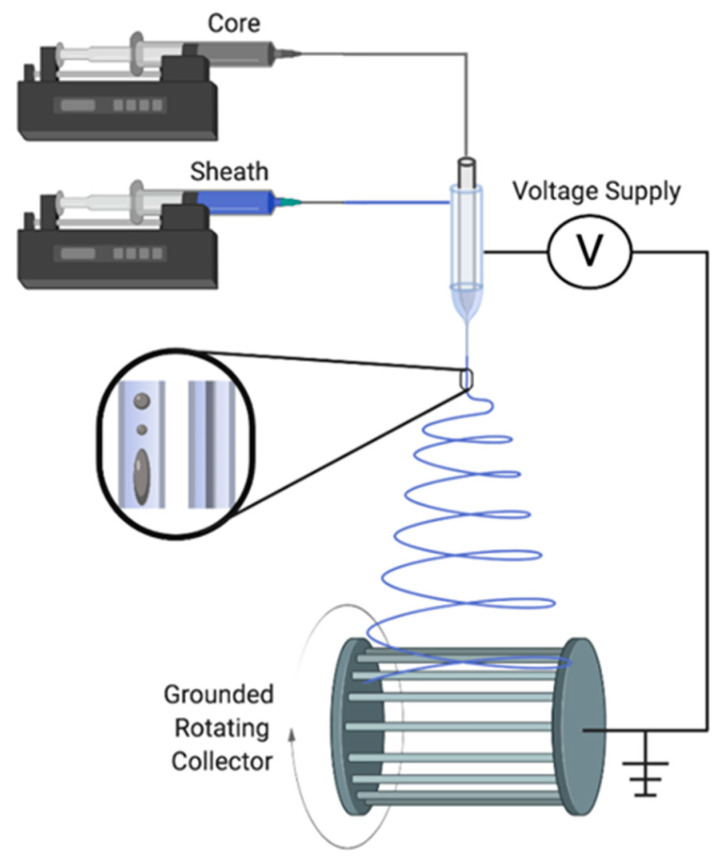
Schematic of the coaxial electrospinning setup.

The electrospinning setup (Figure 5) employed two syringe pumps (Kent Scientific, Torrington, CT, USA) connected using a coaxial electrospinning needle (Ramé-Hart Instrument Co., Succasunna, NJ, USA) with an outer needle gauge of 1.024 mm (18 G) and inner needle gauge of 0.405 mm (26 G). The flow rate varied between 0.6 mL/h and 1 mL/h with the differing compositions. The inner sheath was consistently set at 0.6 mL/h, while the outer sheath varied between 0.8–1 mL/h. The distance between the needle and the collector was between 10–15 cm; 10 cm for the aligned fibers and 15 cm for droplets only. The solution was electrified by applying a voltage between 8–12 kV (CZE1000R, Spellman, Hauppauge, NY, USA), and the experimental setup was spun downwards, as seen in Figure 5. The resulting polymer fibers and droplets were collected on aluminum foil, glass microscope slides or a rotating mandrel.

The monomer/crosslinker/initiator solution in the resulting fibers were polymerized in the oven at 80 °C for 60 min. The PS outer coating was removed by dissolving the PS in toluene in a separation funnel. Water was then added, and the PAA moved toward the lower aqueous layer. This was collected and dried to obtain only the PAA. Other solvents such as tetrahydrofuran and acetone (Fisher) were also used to remove the PS layer on glass microscope slides through several washes of said solvent, which was then washed with DI water.

Optical microscopy images were taken using Nikon Eclipse TS-100F (Tokyo, Japan) in a bright field. FT-IR was conducted using Agilent Cary 630 FTIR (Santa Clara, CA, USA). 

Electrical potentials were measured from PAA superfused in 16 mM KCl with a standard electrophysiological patch-clamp setup. Voltage recordings as a function of time were made by using Ag/AgCl electrodes through a recording micropipette (5–10 MΩ), pulled from thick-walled borosilicate glass (1.5-mm outer diameter, 0.85-mm inner diameter, WPI Sarasota, FL, USA). The resulting fine-tipped (≈2 μ) recording glass micropipettes were then filled with 3 M KCl solution and connected to the amplifier via an Ag/AgCl electrode. Voltage recordings were made with an Axopatch 200B patch-clamp amplifier in current-clamp mode (Molecular Devices, Sunnyvale, CA, USA) and were filtered at 2 kHz (8-pole Bessel). Data were then digitized at 20 kHz into a Windows-PC computer using a Digidata 1550A low noise data acquisition system (Molecular Devices, Sunnyvale, CA, USA) and the PCLAMP 10.5 software suite (Molecular Devices, Sunnyvale, CA, USA). Offline data analysis was carried out with Clampfit 10.5 (part of PCLAMP). 

## Figures and Tables

**Table 1:** **Voltage and PS weight percent in DMF parameters for electrospinning and resulting formation.** Coaxial electrospinning with a core of PAA and distance of 15 cm was used with an outer flow rate of 0.8–1 mL/hr and inner flow rate of 0.6 mL/hr.

Voltage	10 kV	12 kV	15 kV
10% PS	Droplets	Fibers	Particles
12% PS	Droplets	Particles/Fibers	Particles
20% PS	Droplets	Particles/Fibers	Particles

## Data Availability

The data presented in this study are available on request from the corresponding author.
